# The Application of Machine Learning on Antibody Discovery and Optimization

**DOI:** 10.3390/molecules29245923

**Published:** 2024-12-16

**Authors:** Jiayao Zheng, Yu Wang, Qianying Liang, Lun Cui, Liqun Wang

**Affiliations:** 1 School of Pharmacy & School of Biological and Food Engineering, Changzhou University, Changzhou 213164, China; 2 Protein Design Lab, Changzhou AiRiBio Healthcare Co., Ltd., Changzhou 213164, China

**Keywords:** antibody engineering, machine learning, computational antibody design, antibody developability, antibody–antigen interaction

## Abstract

Antibodies play critical roles in modern medicine, serving as diagnostics and therapeutics for various diseases due to their ability to specifically bind to target antigens. Traditional antibody discovery and optimization methods are time-consuming and resource-intensive, though they have successfully generated antibodies for diagnosing and treating diseases. The advancements in protein data, computational hardware, and machine learning (ML) models have the opportunity to disrupt antibody discovery and optimization research. Machine learning models have demonstrated their abilities in antibody design. These machine learning models enable rapid in silico design of antibody candidates within a few days, achieving approximately a 60% reduction in time and a 50% reduction in cost compared to traditional methods. This review focuses on the latest machine learning-based antibody discovery and optimization developments. We briefly discuss the limitations of traditional methods and then explore the machine learning-based antibody discovery and optimization methodologies. We also focus on future research directions, including developing Antibody Design AI Agents and data foundries, alongside the ethical and regulatory considerations essential for successfully adopting machine learning-driven antibody designs.

## 1. Introduction

Antibodies have been broadly used as diagnostic tools and therapeutic drugs in modern medicine thanks to their ability to bind to target antigens. Monoclonal antibodies (mAbs) can provide targeted treatment that modulates immune responses or inhibits specific molecular pathways [1]. In conjugation with small molecular drugs, antibodies can deliver cytotoxic agents directly to diseased cells [2]. More than 130 antibody drugs have been approved by the U.S. Food and Drug Administration (FDA) for clinical use [3]. In 2023, five of the top ten best-selling drugs were antibody therapeutics—pembrolizumab, adalimumab, dupilumab, ustekinumab, and nivolumab [4]. The global antibody drug market is projected to reach more than USD 450 billion by 2030.

Traditional antibody discovery and optimization techniques such as hybridoma technology, phage display, yeast display, B cell cloning, and ribosome display have been used for identifying therapeutic antibodies but are inherently labor-intensive, time-consuming, and costly, often requiring more than six months to yield viable candidates [5]. These traditional methods only investigated a limited antibody space, limiting the possibility of finding optimal antibodies for diagnostic and therapeutic applications. Many computational techniques have been used to help traditional antibody engineering for improving antibody discovery and optimization efficiency. Molecular dynamics simulations, homology-based modeling, and Monte Carlo algorithms have been used to predict antibody structure and guide antibody engineering [6,7,8]. Because of the lack of antibody structure data, these approaches primarily focus on the antibody variable domain.

Recent advancements in protein data availability, GPU technology, and machine learning (ML) can potentially revolutionize antibody discovery and optimization. The abundance of protein structures, interactions, and functional data provides rich datasets for training sophisticated machine learning models, while enhanced computational facility enables efficient execution of complex models and simulations. Machine learning models have been broadly applied in protein research, including neural networks, transformers, and protein language models [9,10]. AlphaFold2 and RoseTTAFold models have achieved high accuracy in predicting protein structures directly from amino acid sequences [11,12]. Generative models such as ProteinBERT, ProteinMPNN, and RFdiffusion have advanced computational protein designs by predicting protein backbones, designing sequences for specific structures, and filtering low-quality protein candidates [13,14,15]. Researchers can use these models to design and optimize proteins with desired properties, such as enzymatic activity and binding. Specialized models focus on antibody design, particularly targeting the complementarity-determining regions (CDRs). IgFold can rapidly predict antibody structures using pre-trained language models and graph neural networks [16], and models like DiffAb can be used for the joint generation of antibody sequences and structures targeting CDR optimization [17]. Additionally, diffusion-based generative models and techniques like variational autoencoders (VAEs) and generative adversarial networks (GANs) accelerate the discovery of novel antibody variants [18,19].

This review focuses on the latest development of machine learning applications in antibody discovery and optimization. We begin by presenting the limitations of traditional antibody discovery methods and computational methods. Then, we introduce the latest ML methodologies, examining how machine learning models are applied to antibody structure prediction, antibody–antigen interaction, and antibody docking. Machine learning innovations for antibody affinity optimization and developability methods were reviewed. Furthermore, we highlight emerging trends, including the potential development of Antibody Design AI Agents and the proposal of Antibody Data Foundry, which emphasizes the generation of wet experiment-based high-throughput antibody mutational data, interaction data, and property data for enhancing antibody machine learning models. The trend of machine learning-driven antibody discovery and optimization methods raises ethical and regulatory concerns on data privacy and algorithmic transparency. Further practical guidance on applying machine learning techniques to antibody design is available in recent studies [20]. Systematic analyses of machine learning-based antibody design and optimization can also be found [21,22].

## 2. Traditional Antibody Discovery and Computational Antibody Discovery Methods

### 2.1. Traditional Antibody Discovery

Traditional antibody discovery methods involve isolating antibodies from animals or humans exposed to specific antigens through techniques such as hybridoma technology, phage display, B cell cloning, and yeast display. Hybridoma technology revolutionized monoclonal antibody production by fusing antibody-producing B cells with immortalized myeloma cells to create hybridomas capable of continuous antibody secretion [23]. This method enables the production of specific monoclonal antibodies but is labor-intensive and relies on animal immunization. The phage display method expresses antibody fragments on the surface of the phage, allowing the selection of antigen antibodies from the phage library [24,25]. Yeast display systems express antibody fragments on the surface of yeast cells, providing a platform for selecting antibodies with high affinity and specificity while ensuring proper folding and post-translational modifications [26,27]. B cell cloning involves isolating antibody-secreting cells from immunized or naturally exposed individuals and using them to produce fully human antibodies with lower immunogenicity risks [28]. This approach can generate fully human antibodies, which have lower immunogenicity risks. These traditional antibody discovery methods are time-consuming and resource-intensive due to animal immunization and several cycles of screens. Traditional antibody discovery methods, such as phage display, are capable of screening millions to billions of antibody sequences, which is often sufficient to identify high-affinity antibodies with favorable developability attributes. However, as screening methods, they only explore a small fraction of the full antibody diversity space. In contrast, machine learning approaches act as design methods, enabling the generation of novel antibody sequences that can access previously unexplored regions of this space, potentially uncovering more optimal candidates. However, exploring new sequence space also comes with unknown risks, including whether these antibodies will retain drug-like properties, favorable developability attributes, and low immunogenicity. Moreover, challenges in antibody development, such as protein stability, protein solubility, protein thermostability, and immunogenicity, pose difficulties in developing antibody candidates for clinical use.

### 2.2. Computational Antibody Engineering Methods

Computational techniques have been used to help traditional antibody discovery and engineering. Molecular dynamics simulation methods imitate the molecular movement of antibodies, which provides information on the dynamic antibody behavior and their interactions with antigens [29,30]. Homology-based modeling is a sequence-aligning-based structure modeling method, which primarily focuses on the modeling of variable regions of antibodies [31,32]. A structure-guided design utilizes computational algorithms to optimize antibody–antigen interactions at the molecular level, enhancing binding affinities and specificities. Numerous antibody Fab structures have been determined experimentally, but very few intact IgG structures are available. Computational methods have therefore focused primarily on the prediction of paired antibody variable domain (VH/VL) structures. Additionally, these methods need heavy computational facilities for antibody simulations.

## 3. Machine Learning for Antibody Discovery

### 3.1. Machine Learning-Based Antibody Structure Prediction

**Figure 1:**
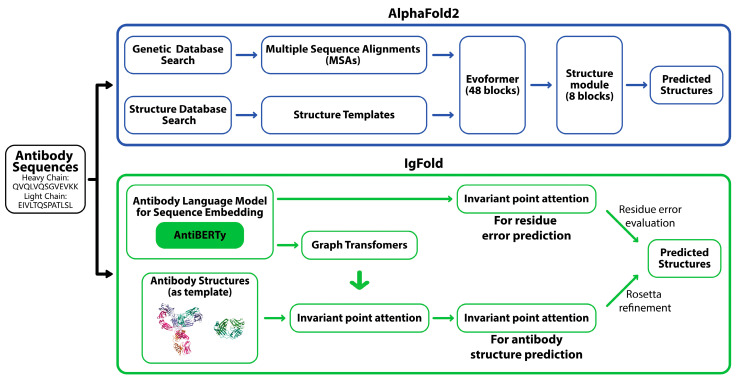
AlphaFold2 and IgFold pipelines.

Models like AlphaFold2 and RoseTTAFold2 have revolutionized protein structure prediction by utilizing deep learning to predict protein structures with high precision. AlphaFold2 pipeline involves generating multiple sequence alignments and identifying homologous structures to capture evolutionary information, which is processed by a deep neural network (Evoformer) to predict inter-residue relationships (Figure 1) [11]. These predictions are then used to assemble and refine a high-accuracy 3D model of the protein. Although AlphaFold models have reinvented general protein structure prediction, antibodies present unique challenges due to their highly variable complementarity-determining regions (CDRs) essential for antigen binding. Specialized models like IgFold have been developed for antibody structure prediction [16]. IgFold leverages embeddings from AntiBERTy, a language model pre-trained on 558 million natural antibody sequences [33], and utilizes graph neural networks to directly predict antibody backbone atom coordinates with remarkable speed and accuracy, completing predictions in under 25 s (Figure 1). IgFold outperforms traditional homology models and matches or surpasses AlphaFold in specific antibody-specific tasks due to the captured intricate residue relationships. IgFold also offers enhanced flexibility, including robust incorporation of template structures and support for nanobody modeling.

The AlphaFold2 pipeline (top) predicts antibody structures by integrating genetic and structure database searches with multiple sequence alignments (MSAs), followed by structural prediction through Evoformer blocks and structure modules. The IgFold pipeline (bottom) employs an antibody-specific approach, utilizing AntiBERTy for sequence embedding. Graph transformers and invariant point attention enable accurate residue error prediction and antibody structure prediction, refined with Rosetta modeling.

ImmuneBuilder is a suite of deep learning models (consisting of ABodyBuilder2, NanoBodyBuilder2, and TCRBuilder2) tailored for accurate and rapid antibody structure prediction [34]. ImmuneBuilder addresses the gap between the abundance of sequence data and the scarcity of antibody structural information. By training models on antibodies, ImmuneBuilder achieves state-of-the-art accuracy while significantly reducing computational time compared to general models like AlphaFold2. For example, ABodyBuilder2 predicts the structure of antibody CDR-H3 loops with an RMSD of 2.81 Å—outperforming AlphaFold-Multimer by 0.09 Å—and does so over a hundred times faster. The models also generate ensembles of structures to provide residue-level error estimates. Both IgFold and ImmuneBuilder are freely accessible, enabling large-scale structural analysis of immune proteins. Additionally, the authors have released millions of predicted paired antibody (paired VH and VL chains) sequences, which are valuable resources to the immunology research community.

### 3.2. Machine Learning-Based Antibody–Antigen Interaction

**Figure 2:**
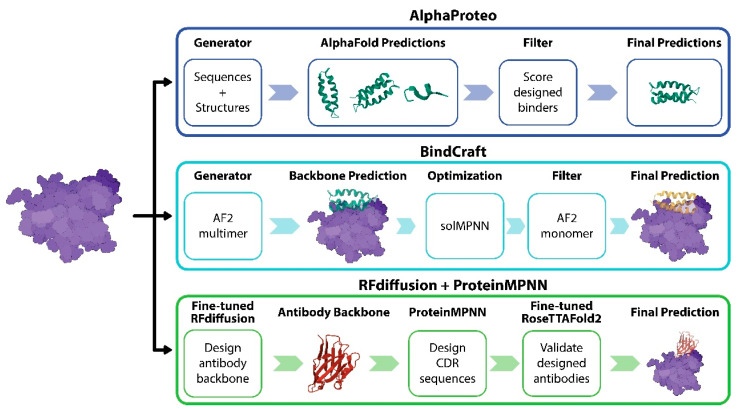
Machine learning models for de binder design and antibody design.

Machine learning models have been used to predict the binder of target proteins. AlphaProteo and BindCraft are leading general protein binder prediction models (Figure 2) [35,36]. AlphaProteo comprises a generative model trained on structural and sequence data from the Protein Data Bank (PDB), a distilled set of AlphaFold predictions, and a filtering mechanism that scores and selects predicted designs (Figure 2). By inputting target protein sequences, AlphaProteo can generate many candidate binders, and then the model filters low-quality binders. The model achieved experimental success rates ranging from 9% to 88%. BindCraft, an open-source and automated protein binder design pipeline, utilized the AlphaFold2 multimer to design high-affinity binders without complicated filtering, even when binding sites are unknown (Figure 2). Based on the 10 experimentally validated binders, BindCraft has a success rate of 10–100%.

AlphaProteo (top) generates protein binders by predicting structures using AlphaFold and scoring designs to filter candidate binders. BindCraft (middle) uses AlphaFold2 multimers and solMPNN (a deep learning model that predicts protein solubility) to generate a backbone and optimize binding, respectively. Then, the AlphaFold2 monomer is used to enhance accuracy for final predictions. The RFdiffusion plus ProteinMPNN approach (bottom) uses a fine-tuned RFdiffusion model to design an antibody backbone and generate CDR sequences, refined with RoseTTAFold2 for structural validation.

The Baker lab explored the de novo design of single-domain antibodies (VHHs) using machine learning tools such as RFdiffusion and ProteinMPNN (Figure 2) [37]. RFdiffusion was fine-tuned to design antibody backbones that can maintain antibody structure. Without immunization and library screens, their method can design nanobody backbones for specific epitopes on antigens. Then, ProteinMPNN was used to optimize the interaction of the complementarity-determining regions (CDRs) region. They have tested this strategy for targets such as influenza, RSV, SARS-CoV-2, and *Clostridium difficile* toxin B. The designed nanobodies were experimentally validated, demonstrating binding to specified epitopes. This study also highlights the potential of using these computational techniques to optimize therapeutic properties such as solubility and aggregation. Even if the success rate of this method is lower than 1%, the method demonstrated promise for rapid and cost-effective antibody discovery, though future efforts will aim to enhance binding affinities and success rates.

Machine learning methods are also enabling the design of antibodies for challenging targets, such as GPCRs. JAM (Joint Atomic Modeling) has successfully designed de novo antibodies targeting these proteins with double-digit nanomolar affinities and promising developability profiles [38]. Additionally, ProteinMPNN and AF2seq have shown the ability to design soluble analogs of membrane proteins, making them accessible for antibody discovery [39]. These examples demonstrate how machine learning is extending the boundaries of what traditional methods can achieve.

### 3.3. Machine Learning-Based Antibody Docking on Antigen

Antibody–antigen docking is a heavy computational process that models the interaction between antibody and antigen. The docking process generates diverse conformations of interacting antibody–antigen complexes and then evaluates and ranks the binding affinities to select the optimal complex. Several models have improved antibody docking accuracy on corresponding antigens (Table 1). GeoDock leverages attention mechanisms and graph-based modules to encode ligand flexibility, achieving a 41% success rate by accounting for conformational adaptability [40]. DLAB improves docking pose ranking using CNNs [41], while dyMEAN, a multi-channel message-passing network [42], surpasses hierarchical models like HERN with a DockQ score of 44% in CDR-H3 docking [43,44]. DockGPT adopts a generative approach, utilizing triangle multiplication and attention to achieve RMSD values between 1.02 Å and 1.88 Å for specific CDR loops [45]. PointDE further improves docking accuracy with 3D point cloud data, reporting a 56.6% success rate by refining docking decoys [46].

The AlphaFold3 presented an innovative framework for protein docking. AlphaFold3 introduces iterative learning, boosting CDR H3 accuracy to 2.04 Å RMSD and achieving an 8.9% success rate for high-accuracy docking [47]. Despite these improvements, AlphaFold3 struggles with a 60% failure rate for single-seed predictions and greatly benefits from antigen context for loops that are longer than 15 amino acids [48,49]. HADDOCK3, on the other hand, employs knowledge-based docking strategies and uses antibody ensembles from models like IgFold and AlphaFold2 to address structural uncertainties [50], which makes it capable of generating near-native antibody–antigen complex models.

## 4. Machine Learning for Antibody Optimization

### 4.1. Machine Learning-Based Antibody Affinity Optimization

Using in silico affinity maturation methods, powered by structure-based modeling and machine learning (ML), has greatly improved the ability to improve antibody binding affinity. Traditional affinity maturation approaches rely on random mutagenesis, which is labor-intensive and slow. Structure-based modeling with free energy calculations plays a crucial role in predicting how mutations impact binding affinity. The GeoPPI model used Graph Attention Networks (GATs) and gradient-boosting trees to predict the change in free energy (ΔΔG) when amino acids are replaced, helping to identify favorable mutations [51]. Another structure-driven deep learning model, GearBind, improved the affinity of CR3022 antibodies by up to 17-fold against the SARS-CoV-2 Omicron strain, demonstrating the power of structural modeling [52].

Deep mutational scanning (DMS) data-trained machine learning models for antibody affinity optimization and selection strategy showed impressive results. For example, an LSTM-based generative model applied to phage display libraries identified sequences with an 1800-fold improved affinity (0.14~900 μM: Parent, 0.0051~33 μM: Best maturated) for anti-kynurenine antibodies [53]. Based on CRISPR-based mutagenesis, a deep neural network trained on trastuzumab variants predicted high-affinity HER2-specific variants [54]. Experimental validation confirmed that all 30 tested variants retained specificity, demonstrating the accuracy of DMS and ML-based approaches in optimizing antibody affinity. Based on yeast display, the MAGMA-seq-generated DMS data facilitates the exploration of binding relationships across diverse antibody libraries by accommodating variations in light chain gene usage, CDR H3 length, and different antigenic targets [55]. Phage display data combined with machine learning enabled the generation of sub-nanomolar affinity antibody libraries [56]. Machine learning techniques and deep mutational scanning (DMS) data enabled the systematic identification of critical antibody development pathways, key paratope sequence determinants, and precise binding epitopes.

### 4.2. Machine Learning-Based Developability Optimization

#### 4.2.1. Predicting Antibody Biophysical Properties

Antibody aggregation propensity and poor solubility can decrease therapeutic efficacy and trigger immunogenic responses. Machine learning models trained on sequence-based features, such as amino acid composition, hydrophobicity, and structural motifs, can predict aggregation-prone regions in antibody sequences [57]. For example, molecular hydrophobicity and surface charge distribution data can be extracted to build predictive models. Spatial positive charge mapping on the CDRH2 loop and solvent-accessible hydrophobic areas on the variable fragment have been shown to correlate strongly with aggregation rates, achieving high predictive performance using k-nearest neighbor (KNN) models (r = 0.89) [58]. Antibody solubility issues can be alleviated by using machine learning models trained with antibody net charge data. These models can suggest sequence mutations to mitigate aggregation risks.

Antibody thermostability, which can influence both biological function and manufacturing outcomes, depends heavily on the antibody structural integrity and flexibility of variable regions, yet traditional predictive methods based on sequence or static structural models fall short due to their inability to capture entropic information. AbMelt integrates high-temperature molecular dynamics (MD) simulations with machine learning (ML) to predict thermostability metrics, including aggregation temperature, onset of melting temperature, and melting temperature [59]. The deviation of internal contacts at 350 K shows a strong Pearson correlation with both the onset of melting temperature (rp = −0.74) and melting temperature (rp = −0.69), suggesting that the capacity of antibodies to maintain structural integrity under thermal stress is a critical factor for stability. AbMelt outperforms traditional models by predicting thermostability endpoints with high accuracy on test sets, achieving R^2^ values above 0.56. These findings demonstrate the utility of molecular dynamics simulation to capture features, which are used to build an antibody thermostability predictive model. However, current machine learning models for antibody biophysical property prediction primarily focus on single parameters, which may be due to the lack of large-scale, highly integrated datasets for antibody biophysical parameters.

#### 4.2.2. Machine Learning Models for Immunogenicity Prediction

Immunogenicity can induce anti-drug antibodies, which are triggered by B cell and T cell activation, compromising drug efficacy and causing severe complications. Wet lab experiments for immunogenicity assessment are costly and time-consuming, making in silico approaches much more favorable. Predictive models analyze amino acid motifs to identify epitopes that are likely to bind major histocompatibility complex (MHC) molecules, informing the design of antibodies with reduced immunogenicity. Databases such as the Immune Epitope Database (IEDB) and Open Antibody Space (OAS) have enabled machine learning (ML) models to predict immune responses more in silico [60]. The antibody language model, AntiBERTy, which has been pre-trained on antibody sequences, has a balance between immunogenicity reduction and functional preservation [33].

Building on AntiBERTy’s capabilities, the AbImmPred framework offers a more refined immunogenicity prediction method based on the variable regions of antibodies [61]. Using AntiBERTy for feature extraction and Principal Component Analysis (PCA) for dimensionality reduction, AbImmPred trained an ensemble model through the AutoGluon automated machine learning framework. Trained on a dataset of 199 therapeutic antibodies, AbImmPred achieved an accuracy of 0.7273 on independent test data. This model also demonstrated better precision, recall, and F1-score, showing its robustness and practical utility for immunogenicity screening.

## 5. Opportunities and Challenges

Machine learning has achieved remarkable advancements in antibody discovery and optimization. Tools like AlphaFold2 and its antibody-specific extensions, such as IgFold, have enabled protein structure prediction by achieving high precision and rapid processing. These models can accurately predict antibody structures, including the challenging complementarity-determining regions (CDRs), within minutes, enabling large-scale antibody design and structural analysis. Additionally, methods like AlphaProteo and BindCraft have successfully generated high-affinity binders for specific targets, demonstrating machine learning’s capability to navigate the antibody design spaces efficiently.

**Figure 3:**
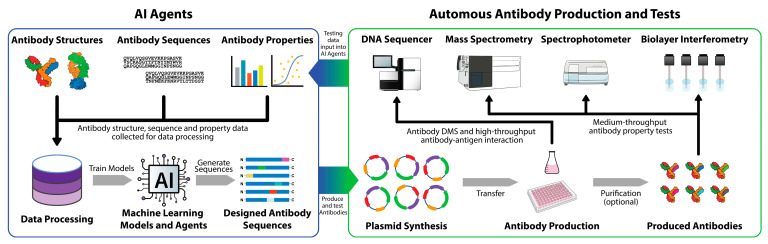
Antibody Design AI Agent and autonomous antibody production and testing.

The integration of machine learning with autonomous systems and robust datasets holds the potential to further accelerate and optimize antibody discovery (Figure 3). The development of an Antibody Design AI Agent envisions a collaborative multi-agent system capable of end-to-end optimization, integrating tasks like antibody generation, property evaluation, and iterative refinement. Such systems can leverage ML models like RFdiffusion and ProteinMPNN, coupled with high-throughput experimental data, such as DMS, to create antibodies optimized for multiple parameters, such as binding affinity, developability, and low immunogenicity risks.

### 5.1. Antibody Design AI Agent

The integration of machine learning (ML) agents with robotics presents the opportunity to automate and accelerate the process of drug development [62]. Drawing from the concepts of SAMPLE (a self-driving laboratory platform for protein landscape exploration) and ProtAgents (a multi-agent framework for protein design), we propose the future opportunity of Antibody Design AI Agents [63,64]. This system would act as an autonomous collaborator, capable of end-to-end antibody optimization by iteratively designing, testing, and refining candidates based on key biophysical properties like binding affinity, solubility, expression, thermostability, and immunogenicity. Ideally, the AI agents would operate in an autonomous improvement loop, using predictive models to explore the fitness landscape and, in parallel, experimental feedback to improve antibody design models.

The Antibody Design AI Agent would combine state-of-the-art antibody design models, antibody optimization models, and high-throughput experimental workflows to explore antibody structure and sequence spaces efficiently and autonomously. Multiple specialized antibody design agents within the system would collaborate. The antibody design will start from antibody generative models, such as BindCraft, AlphaProteo, and IgFold, which can generate novel antibodies that meet multi-objective constraints, ensuring reasonable antibody fitness. The proposed sequences would pass through specialized sub-agents, such as expression and evaluation agents, which express and evaluate antibodies on a medium-throughput scale (500–1000), where antibody property data (expression, binding affinity, aggregation, thermostability, immunogenicity, and poly-reactivity) are collected. The experimental results from expression and evaluation tests would feed back into the antibody generative models, enabling it to improve its ability to predict better antibodies. This multiple Antibody Design AI Agent system could automate complex decision-making with different tasks. One agent might specialize in retrieving knowledge from the scientific literature to suggest substitutions to minimize aggregation risks, while another uses physics-based simulations to evaluate structural integrity. The collaboration among agents would ensure a synergistic optimization process, where distinct agents collaboratively solve de novo antibody design tasks by integrating machine learning models and molecular dynamics simulations.

This schematic represents an end-to-end, machine learning agent-powered autonomous platform for antibody design, production, and testing. AI agents trained to generate optimized antibody candidates process antibody structures, sequences, and property data. High-throughput experimental systems (DNA sequencers, mass spectrometry, spectrophotometers, and biolayer interferometry) test antibody properties, which feed back into AI models for further optimization. Antibody production and purification systems allow for scalable synthesis and testing of AI agent-generated antibodies, facilitating an iterative pipeline that accelerates discovery and optimizes binding and developability.

The Antibody Design AI Agent holds the potential to accelerate therapeutic antibody discovery by autonomously balancing multiple developability parameters. This system would improve antibody design efficiency and reduce the cost of wet experiments for human resources. The agent could also include coupling the platform with cloud laboratories for global accessibility, enabling continuous, data-driven antibody development in real-time. Such an approach has the potential to transform therapeutic antibody pipelines, delivering safer and more effective biologics at unprecedented speeds.

### 5.2. Antibody Data Foundry

The development of robust machine learning models for antibody design relies on high-quality, comprehensive datasets. Current antibody data are fragmented and lack of reproducibility. Here, we propose the idea of an Antibody Data Foundry, an integrated platform comprising three distinct components of data.

The first component uses experimental and computational methods to generate protein structure and sequence data. This includes generating high-resolution antibody structures using crystallography, cryo-electron microscopy, and AlphaFold-based predictions. Novel antibody sequence data will be generated via mutational libraries, guided by generative models like AntiBERTy or DiffAb. These data will help to broaden the exploration of the antibody fitness landscape and capture structural and functional diversity. The second component leverages existing public databases, such as OAS (over 2 billion immune sequences), SAbDab (curated antibody structures), and NanoLAS (specializing in nanobody data), to provide foundational data on antibody sequences, structures, and interactions. Current antibody structure prediction and design models were built based on those available antibody databases.

The third component focuses on generating and aggregating high-throughput experimental data critical for enhancing antibody design and optimization. This includes real-world experimental datasets of high-throughput antibody–antigen interaction dynamics [65], antibody deep mutational scanning [66], and high-throughput antibody property data, often underrepresented in current repositories but essential for model improvement and optimization. The foundry will collect and standardize these data, enabling the training of improved antibody design models. With the Antibody Data Foundry in place, the field of computational antibody design will gain antibody discovery and optimization efficiency, and a more reliable development of therapeutics, addressing both the practical and computational challenges currently limiting progress.

### 5.3. Ethical Considerations and Regulatory Compliance

Machine learning antibody design methods offer new opportunities but also pose challenges. Ensuring patient safety and therapeutic efficacy requires strict compliance with standards from regulatory agencies such as the U.S. Food and Drug Administration (FDA), the European Medicines Agency (EMA), and the National Medical Products Administration (NMPA). In 2023, the FDA published a white paper on using artificial intelligence and machine learning in the development of drug and biological products [67]. Data privacy and ownership are key ethical concerns, especially when models utilize patient-derived sequences or clinical data [68]. Compliance with data protection laws like the General Data Protection Regulation (GDPR) is essential to safeguard sensitive information. Transparent data practices describing how data are collected, stored, and applied are critical for maintaining trust and ensuring data reproducibility. Developing explainable models will enhance trust among regulators, clinicians, and researchers. For medical devices, the U.S. Food and Drug Administration (FDA), Health Canada, and the United Kingdom’s Medicines and Healthcare Products Regulatory Agency (MHRA) have jointly published 10 guidelines for good machine learning practice (GMLP) [69]. Additionally, biases in training datasets, such as the underrepresentation of certain antibody classes or rare targets, can limit model generalizability and should be mitigated through curated, diverse datasets [70]. Ensuring model reproducibility and transparency requires standardized reporting of data preprocessing, model architectures, and evaluation metrics, as well as open access to training data and source code where feasible. We expect and encourage regulatory bodies to publish good practice guidelines for the application of machine learning to drug design. Collaborative efforts between researchers, ethicists, and regulatory agencies will be essential to establish ethical guidelines and best practices, aligning machine learning methodologies with regulatory expectations.

## 6. Conclusions

Advances in machine learning-based structure prediction models, antibody–antigen interaction modeling, and optimization tools have accelerated antibody discovery and optimization. General and specialized models like AlphaFold and IgFold have significantly improved their ability to predict antibody structures, which enormously increases the availability of antibody structures. General protein binder prediction models, such as AlphaProteo and BindCraft, showed high antibody design accuracy. Specialized antibody design models based on RFdiffusion and protineMPNN were developed for antibody design with reasonable accuracy. With the help of machine learning for antibody discovery and optimization, it is possible to generate a viable antibody candidate within 1–2 months, compared to the 6–9 months required by traditional methods. This represents a 60% reduction in the time required for the initial stages of antibody candidate generation [20].

Despite rapid development in this area, there is a strong need for high-quality, comprehensive antibody datasets; also, there are concerns regarding ethical and regulatory considerations. The proposed Antibody Data Foundry can increase the availability of integrated and standardized antibody training data, which is key for improving machine learning-based antibody discovery and optimization. Opportunities such as developing autonomous systems like the Antibody Design AI Agent hold promise for automating and accelerating antibody development even further. In conclusion, integrating machine learning into antibody research accelerates the discovery process. It opens new frontiers in therapeutic development, potentially delivering safer and more effective biologics at unprecedented speeds.

## Figures and Tables

**Table 1:** Overview of machine learning models for protein and antibody docking.

Name	Class	Model	Docking Score	Disadvantages
GeoDock	Protein	Transformer	SSR: 41%	Relies on transformer models that can struggle with large, highly diverse datasets.
dyMEAN	Antibody	MEAN	DockQ: Full: 41.2% CDRs: 39.6% H3: 40.9%	Performance on docking antibody CDRs and H3 regions is relatively low, with limited generalization across diverse antibody–antigen interfaces.
DockGPT	Antibody	Transformer	DockQ: 26.1%	Exhibits low DockQ scores compared to other models, indicating challenges in achieving accurate docking for complex antibody–antigen interactions.
PointDE	Protein	PMLP	SSR protein: 65.6% Ab-Ag: 56.6%	Only use PDB file.
AlphaFold3	Protein	Pairformer	Antibody: 8.9% Nanobdy: 13.4%	Low DockQ scores for antibodies (8.9%) and nanobodies (13.4%), reflecting limited docking precision compared to its structural prediction capabilities.

## Data Availability

All relevant data are within the article.
